# Practical Lessons on 12-Lead ECG Classification: Meta-Analysis of Methods From PhysioNet/Computing in Cardiology Challenge 2020

**DOI:** 10.3389/fphys.2021.811661

**Published:** 2022-01-14

**Authors:** Shenda Hong, Wenrui Zhang, Chenxi Sun, Yuxi Zhou, Hongyan Li

**Affiliations:** ^1^National Institute of Health Data Science, Peking University, Beijing, China; ^2^Institute of Medical Technology, Peking University Health Science Center, Beijing, China; ^3^Department of Mathematics, National University of Singapore, Singapore, Singapore; ^4^Key Laboratory of Machine Perception (Ministry of Education), Peking University, Beijing, China; ^5^School of Electronics Engineering and Computer Science, Peking University, Beijing, China; ^6^School of Computer Science and Engineering, Tianjin University of Technology, Tianjin, China; ^7^RIIT, TNList, Department of Computer Science and Technology, Tsinghua University, Beijing, China

**Keywords:** electrocardiogram, machine learning, deep learning, classification, practical lessons, physionet challenge, meta-analysis

## Abstract

Cardiovascular diseases (CVDs) are one of the most fatal disease groups worldwide. Electrocardiogram (ECG) is a widely used tool for automatically detecting cardiac abnormalities, thereby helping to control and manage CVDs. To encourage more multidisciplinary researches, PhysioNet/Computing in Cardiology Challenge 2020 (Challenge 2020) provided a public platform involving multi-center databases and automatic evaluations for ECG classification tasks. As a result, 41 teams successfully submitted their solutions and were qualified for rankings. Although Challenge 2020 was a success, there has been no in-depth methodological meta-analysis of these solutions, making it difficult for researchers to benefit from the solutions and results. In this study, we aim to systematically review the 41 solutions in terms of data processing, feature engineering, model architecture, and training strategy. For each perspective, we visualize and statistically analyze the effectiveness of the common techniques, and discuss the methodological advantages and disadvantages. Finally, we summarize five practical lessons based on the aforementioned analysis: (1) Data augmentation should be employed and adapted to specific scenarios; (2) Combining different features can improve performance; (3) A hybrid design of different types of deep neural networks (DNNs) is better than using a single type; (4) The use of end-to-end architectures should depend on the task being solved; (5) Multiple models are better than one. We expect that our meta-analysis will help accelerate the research related to ECG classification based on machine-learning models.

## 1. Introduction

Cardiovascular diseases are one of the leading causes of death worldwide (Virani et al., 2021). Electrocardiogram (ECG) is the most representative and important non-invasive tool for diagnosing cardiac abnormalities (Kligfield, 2002). The effectiveness of using a standard 12-lead ECG for the diagnosis of various cardiac arrhythmias and other diseases has been proven in several studies (Kligfield et al., 2007). Owing to the predictability of ECG for short-term and long-term mortality risks (Raghunath et al., 2020), accurate and timely detection of cardiac abnormalities based on 12-lead ECG can significantly help save people's lives (Virani et al., 2021). However, manual interpretation of ECG is time-consuming, and different cardiologists may disagree on complicated cases (Hannun et al., 2019; Ribeiro et al., 2019).

In recent years, machine-learning methods have been employed to rapidly detect cardiac abnormalities in 12-lead ECGs (Ye et al., 2010; Jambukia et al., 2015; Minchole et al., 2019; Al-Zaiti et al., 2020). Newly emerging deep-learning models have further achieved comparable performance to clinical cardiologists on many ECG analysis tasks (Hannun et al., 2019; Hong et al., 2020b; Sinnecker, 2020; Elul et al., 2021; Somani et al., 2021), such as cardiovascular management (Fu et al., 2021; Siontis et al., 2021) and arrhythmia/disease detection (Attia et al., 2019; Erdenebayar et al., 2019; He et al., 2019; Hong et al., 2019a,b, 2020a; Zhou et al., 2019; Raghunath et al., 2020; Ribeiro et al., 2020). However, as high-quality real-world ECG data is difficult to acquire, most deep-learning models are designed to detect only a small fraction of cardiac arrhythmias, owing to the limitations of the datasets.

**Figure 1:**
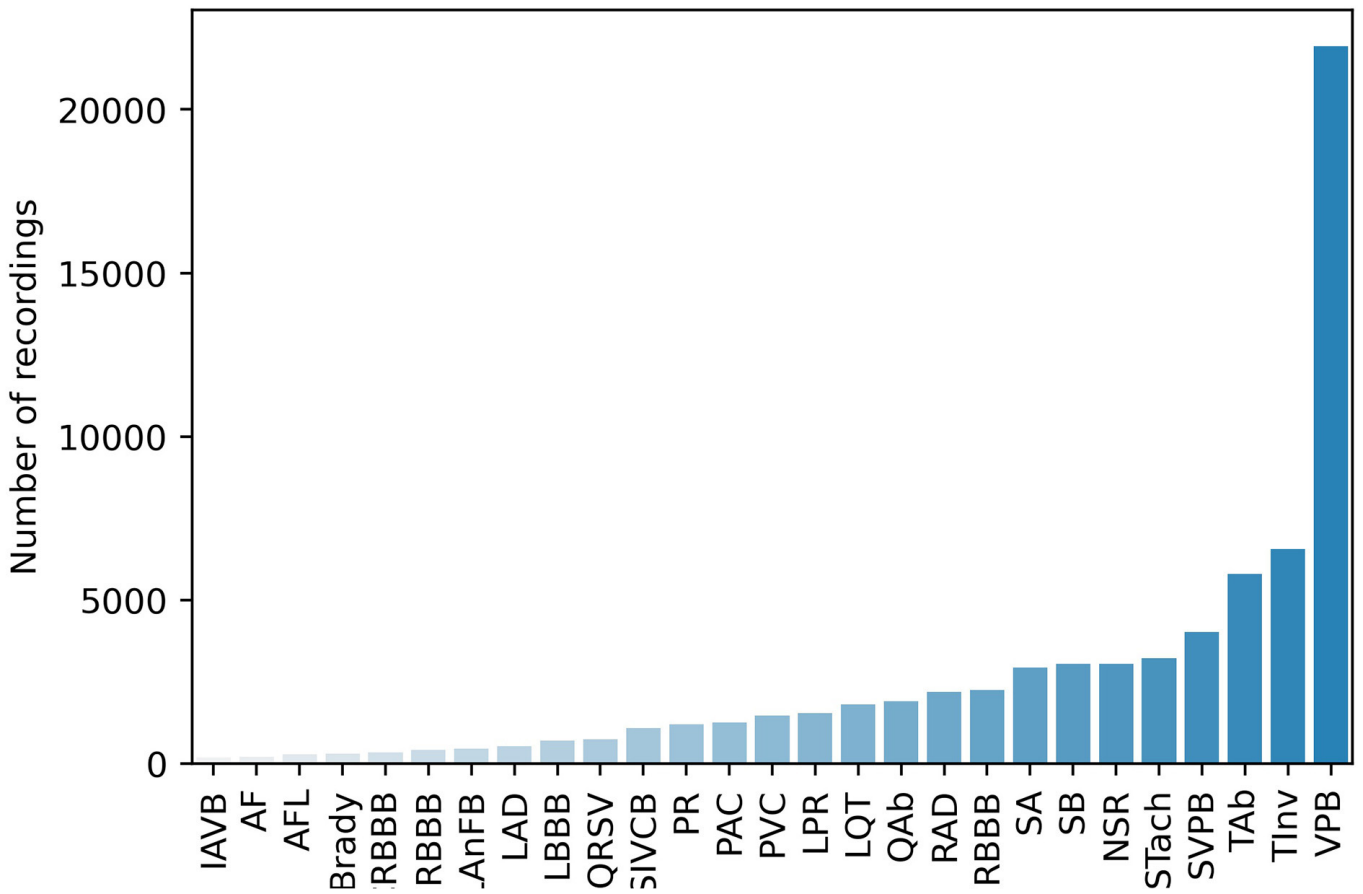
Number of recordings of each scored diagnosis.

PhysioNet/Computing in Cardiology Challenge 2020 (Challenge 2020) provided high-quality 12-lead ECG data obtained from multiple centers with a large set of cardiac abnormalities (Goldberger et al., 2000; Alday et al., 2020; PHY, 2020; Raghunath et al., 2020). The aim of Challenge 2020 was to identify clinical diagnoses from 12-lead ECG recordings, providing an opportunity to employ various advanced methods to address clinically important questions that are either unsolved or not well-solved (Alday et al., 2020). The datasets for Challenge 2020 were sourced from multiple medical centers worldwide. As shown in Table 1, all the datasets contain recordings, diagnostic codes, and demographic data. There are 66,361 ECG recordings, and the number of diagnostic classes is 111. As shown in Figure 1, 27 diagnoses are included to evaluate the methods by using an evaluation metric designed by Challenge 2020. This evaluation metric assigns different weights to different classes based on the harmfulness of misdiagnosis in the clinic. The unnormalized challenge score is the summation of the element-wise dots of the confusion matrix and a given reward matrix.

**Table 1:** Overview of databases used in Challenge 2020.

**Database**	**Total**	**Recordings in**	**Recordings in**	**Recordings in**	**Total**
	**Patients**	**Training set**	**Validation set**	**Test set**	**Recordings**
CPSC (Liu et al., 2018)	9,458	10,330	1,463	1,463	13,256
INCART (Tihonenko et al., 2008)	32	74	0	0	74
PTB (Bousseljot et al., 1995; Wagner et al., 2020)	19,175	22,353	0	0	22,353
G12EC (G12, 2020)	15,742	10,344	5,167	5,167	20,678
Undisclosed	Unknown	0	0	10,000	10,000
Total	Unknown	43,101	6,630	16,630	66,361

**Figure 2:**
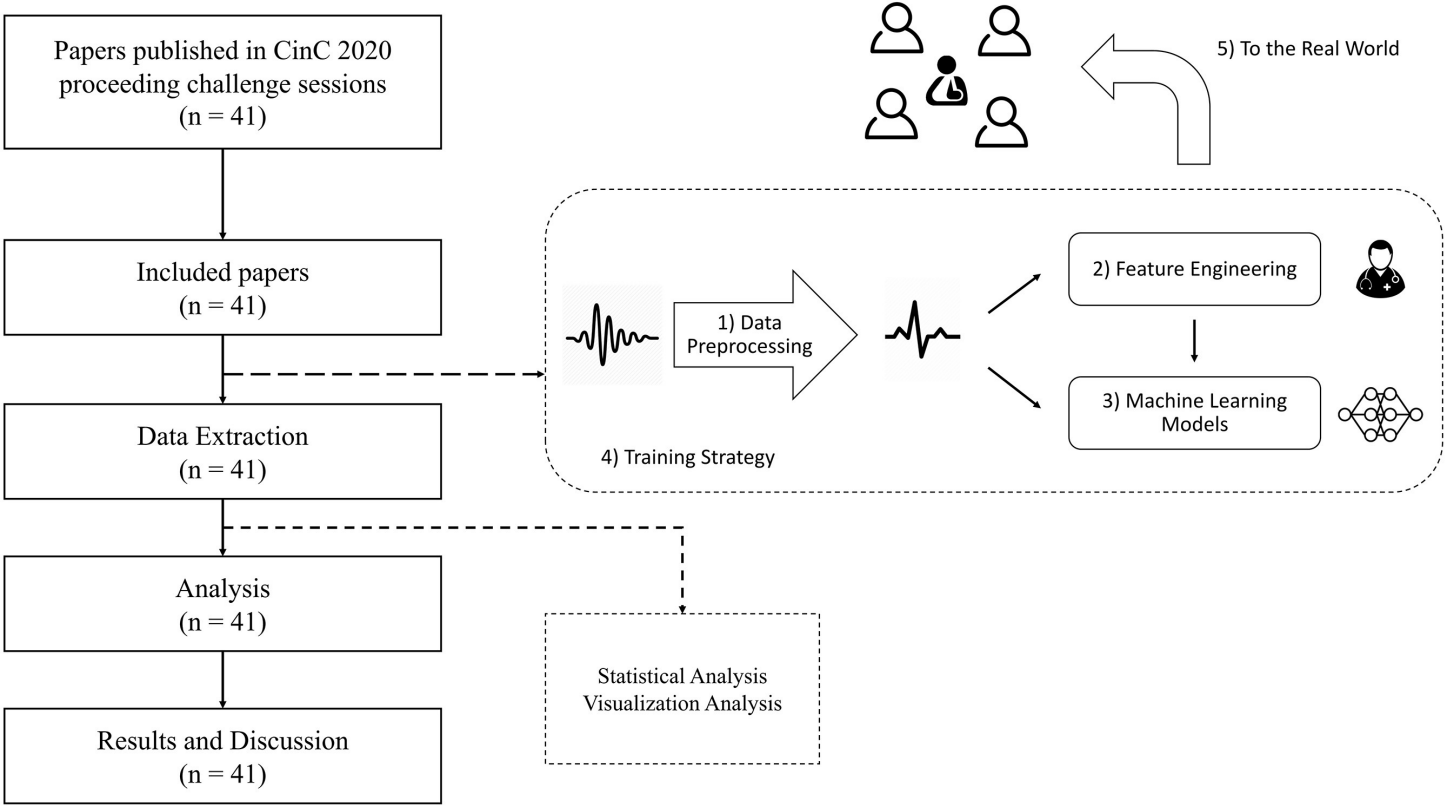
The framework of our meta-analysis.

Many well-designed methods were proposed in Challenge 2020. To obtain a comprehensive understanding of how these methods benefit automated ECG interpretation, a more systematic analysis is needed to compare the differences and similarities among them. Thus, in this study, we conduct a meta-analysis of the 41 methods that qualified to be in the final rankings. We analyze the methods in terms of five aspects: data processing, feature engineering, machine-learning models, training strategy, and applications to the real world (see Figure 2). Through our meta-analysis, we gather the details of the five aforementioned aspects and conduct the Mann-Whitney *U*-test to verify the effectiveness of the methods. Finally, we discuss the reasons for the effectiveness or ineffectiveness of the methods and summarize five practical lessons that can be applied in real-world scenarios or scholarly research.

Our main practical lessons are the following:

1. Data augmentation should be employed and adapted to specific scenarios.
2. Combining different features can improve performance.
3. A hybrid design of different types of deep neural networks (DNNs) is better than using a single type.
4. The use of end-to-end architectures should depend on the task being solved.
5. Multiple models are better than one.

## 2. Method

### 2.1. Search Strategy and Inclusion Criteria

In Challenge 2020, 70 teams successfully implemented their methods on the platform's test data. We conduct our analysis for the 41 teams that qualified to be on the final rankings of the Computing in Cardiology (CinC) conference^1^. The reasons for the disqualification of the other 29 teams are the following: the method did not work on the hidden set, the team failed to submit a preprint or a final article on time, or the team was absent in CinC.

### 2.2. Data Extraction

To investigate the techniques applied by each team, we considered five aspects of the methods that formed a solution pipeline (see Figure 2): *data preprocessing, feature engineering, machine-learning models, training strategy*, and *applications to the real world*. Table 2 presents these five aspects.

**Table 2:** Details of employed techniques.

**Aspect**	**Inclusion**	**Usage(%)**	**# in top-10**	***p*** **-value**
			**methods**	
Data preprocessing	Signal processing	95.12	10	N.A.
	Data augmentation	31.70	6	0.071
	Imbalance handling	53.66	7	0.252
Feature engineering	Hand features	36.59	0	0.983
	Demographic features	29.27	5	0.109
Machine-learning models	Deep neural network	82.93	10	0.116
	Convolutional neural network	82.93	10	0.116
	Recurrent neural network/transformer	31.71	4	0.317
	Attention	24.39	6	0.006
Training strategy	Model ensemble	36.59	4	0.878
	End-to-End	80.49	10	0.139
	Multi-binary classification	58.54	10	0.002
Applications to the real world	Post-processing	2.38	1	N.A.
	Interpretability	4.76	0	N.A.
	Unknown classes and unseen patients	0	0	N.A.

*N.A. means that the hypothesis test is not conducted*.

We confirmed whether a team used a specific technique in their solution by using a three-step reading and checking strategy. First, each reviewer carefully read the full text of 41 papers and extracted data for a single aspect. The data includes whether or how the teams employ techniques involved in the aspect. If a technology is not mentioned in a paper, we assumed that the corresponding team did not use that technology. All the results were gathered together and summarized in a spreadsheet file. Second, each reviewer checked the whole spreadsheet and added comments on what they disagreed with. Finally, all the reviewers discussed the disagreements and corrected the mistakes in the spreadsheet. Thus, we reached the final spreadsheet, and this spreadsheet can be found at https://github.com/hsd1503/cinc2020_meta.

### 2.3. Analytic Approach

Our analytic approach consists of three main steps. First, for each technique mentioned in Table 2, we calculated the usage percentage of the method of the 41 teams. Then, we collected official scores on the test set of each team, and grouped teams based on whether they employed a specific technique. Finally, we statistically analyzed whether these techniques are useful for ECG classification. The commonly used student *t*-test requests that the data follows the normal distribution. However, the distribution is unknown. So we adopt the Mann Whitney *U*-test, a more general and also widely used statistical test method. We conducted the Mann-Whitney *U*-test (Mann and Whitney, 1947) using SciPy library version 1.6.2^2^ and Python version 3.8.8 for each technique. An alternative hypothesis is that the treated technique can improve the performance of the model. We combined two groups, sorted them in ascending order, and assigned ranks for samples (the smallest sample is set as 1, the second smallest sample as 2, and so on). We calculated the sum of the ranks of the two groups referred to as *R*_1_ and *R*_2_. The U-statistics are computed as


(1) $$\begin{array}{r} U_{i}=R_{i}-\frac{n_{i}\left(n_{i}+1\right)}{2},i=1,2 \end{array}$$

where *n*_*i*_ is the number of samples in the i-th group. Then, we let *U* = 3*U*_1_ because our alternative hypothesis is that the values of group 1 are statistically larger than those of group 2. The Z-statistics are computed as


(2) $$\begin{array}{r} Z=\frac{U-\frac{n_{1}\times n_{2}}{2}-0.5}{\sqrt{\frac{n_{1}\times n_{2}}{12}\times \left(\left(n_{1}+n_{2}+1\right)-\frac{tie}{\left(n_{1}+n_{2}\right)\times \left(n_{1}+n_{2}-1\right)}\right)}}, \end{array}$$

(3) $$\begin{array}{r} tie=\overset{n_{1}}{\underset{i=1}{\sum }}count\left(group_{1i}\right), \end{array}$$

where *count*(*group*_1*i*_) represents the number of values in groups 1 and 2, equal to the i-th value in group 1. The *p*-value is


(4) $$\begin{array}{r} p=P\left(x>Z\right),x\sim N\left(0,1\right). \end{array}$$

To better visualize the results, we drew box plots for each technique. The box figures show groups for the median, upper quartile, lower quartile, outliers, and range of official scores on the test set. In addition, we discussed and explained why some techniques are beneficial and explore practical lessons from the methods in Challenge 2020.

## 3. Results

### 3.1. Overview

The overall meta-analysis results are listed in Table 2. We can observe that some techniques are used by the majority of the teams. The results indicate that ECG classification is a complex process that includes multiple techniques. Among these techniques, signal processing, DNNs, convolutional neural networks (CNNs), end-to-end and multi-binary classifications are used by all of the top 10 teams. In addition, we have several significant findings: 1) deep-learning methods were more popular than traditional methods in Challenge 2020; 2) all the teams that employed deep-learning methods used CNNs; and 3) none of the top-10 teams used hand-labeled features (except demographic features); they all adopted end-to-end models instead.

### 3.2. Data Preprocessing

In this section, we focus on three components of data preprocessing: *signal processing, data augmentation*, and *imbalance handling*.

#### 3.2.1. Signal Processing

Signal processing is the most common technique used for ECG classification. We did not attempt to verify the effect of signal processing because different teams set different sampling rates and window sizes and applied various methods. Instead, we summarize and discuss the most common signal processing techniques used in Challenge 2020: resampling, resizing, filtering, and normalization.

Resampling aims to eliminate the differences in the sampling rates among the different input samples. This is necessary because varied sampling-rate inputs degrade the classification models. In the real world, ECG recordings are collected from various medical devices with different sampling rates. Training machine-learning models with this type of data is difficult because their data distributions are inconsistent. This problem can be solved by interpolating the data to a unified sampling rate. Resizing is often realized by cutting signals into a fixed length (known as the window size). Resizing also aims to satisfy another common training request: that the length of the training samples should be the same. Filtering, usually by using band-pass filters, is applied to denoise raw signals. This prevents the model from being disturbed by noise, and this can usually improve performance. Normalization standardizes the signals to a normal distribution or even distribution by transforming signal values in the range of [0, 1] or [-1, 1]. Data distributions can be unified and the influence of noise and outliers can be alleviated through normalization. Other signal processing techniques such as zero-padding (Natarajan et al., 2020), median filters (Hsu et al., 2020), and wavelet transformation denoising (Zhu et al., 2020) can also be used.

#### 3.2.2. Data Augmentation

Data augmentation is an efficient tool for increasing the size and enhancing the quality of the training data. It mainly aims to generate more data covering unseen input spaces (Wen et al., 2020). Data augmentation can make the model more robust by enlarging the size and adding noise or causing transformation. This is also an effective method to avoid overfitting. Common data augmentation methods in Challenge 2020 included the introduction of external data (Bos et al., 2020; Zhu et al., 2020), addition of noise (Chen et al., 2020; Weber et al., 2020), and random cropping (Duan et al., 2020a; Weber et al., 2020). All these methods enlarged the size of the training data. However, when augmentation is performed, the extent of augmentation (such as the stride of the sliding window augmentation) must be considered. Augmenting too much may destroy the distribution of data and cause failure in learning common patterns in data.

**Figure 3:**
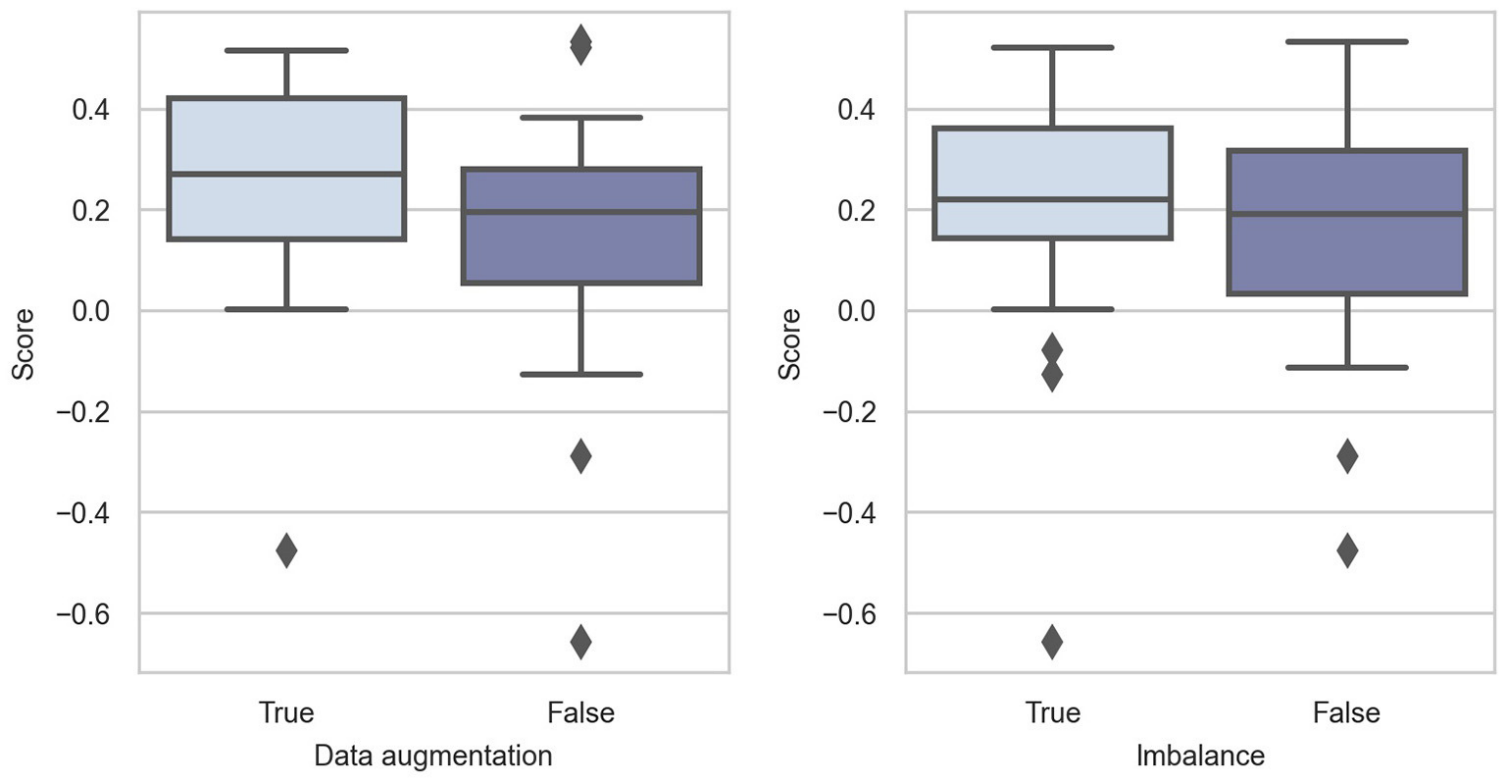
Box-plots of score distributions of data preprocessing techniques.

In Figure 3, we can see that data augmentation is intuitively beneficial in Challenge 2020. All descriptive statistics are larger when data augmentation is performed. The *p*-value of the Mann-Whitney *U*-test is 0.07, which is slightly larger than 0.05 without data augmentation. As the sample size is small, we believe that the alternative hypothesis holds.

#### 3.2.3. Imbalance Handling

The training data in Challenge 2020 suffer from heavy class imbalance (as shown in Figure 1), which results in predictions being biased toward the majority classes. This is because the training samples of the majority class dominate in the training phase, and they bias the model objectives so that it is easier to obtain higher overall accuracy. In addition, classes with minority sample sizes are more difficult to learn. Even when a classification model is successfully trained, it would very likely become an over-fitted model. Therefore, solving this problem also significantly affects model performance. As shown in Figure 3, handling class imbalance can improve the performance of the models.

In Challenge 2020, teams attempted to overcome this problem in two main ways: threshold optimization (Chen et al., 2020; Fayyazifar et al., 2020; Zhao et al., 2020) and weighted loss (Bos et al., 2020; Min et al., 2020). Threshold optimization aims to select the appropriate thresholds corresponding to each class; this has proven to be feasible (Kang et al., 2019). This method is based on models preferring to output a high probability for major classes; thus, setting a low threshold for minor classes can help alleviate this problem. Loss weights are assigned for each class, and the weighted loss forces each class to contribute equally to training the model. In addition, over-sampling (Zisou et al., 2020), down-sampling (Hsu et al., 2020), and other methods have been employed in Challenge 2020.

### 3.3. Feature Engineering

In this section, we examine how the teams choose or engineer features for model inputs in terms of two aspects: *hand features* and *demographic features*.

#### 3.3.1. Hand Features

In our analysis, we regard hand features as features extracted through non-machine-learning methods, while not simply selecting raw features such as age and sex. Hand features can be further divided into temporal and frequent features.

1. **Temporal Features**: Temporal features are related to the morphological characteristics of ECG waves. The extraction of temporal features consists of two steps: wave detection and measurement computing. In Challenge 2020, teams usually employed traditional waves detection methods, such as P-wave, QRS-complex, and T-wave, and then explicitly computed ECG measurements as feature vectors, such as P-wave duration, PR interval, QRS duration, and ST slope. The details of the temporal features can be found in Hong et al. (2019b). These ECG-specific features have proven to be effective for the diagnosis of cardiac diseases.
2. **Frequent Features**: Frequency domain is also an important part of ECG hand features. Thus, some teams extracted features focusing on the frequency spectrum, excluding temporal information. The frequency domain helps to inspect signals from a different view rather than only from the temporal domain. For example, the frequency bands of 0.67–5 Hz, 1–7 Hz, and 10–50 Hz are commonly considered as the dominant components of P-wave, T-wave, and QRS-complex, respectively.

**Figure 4:**
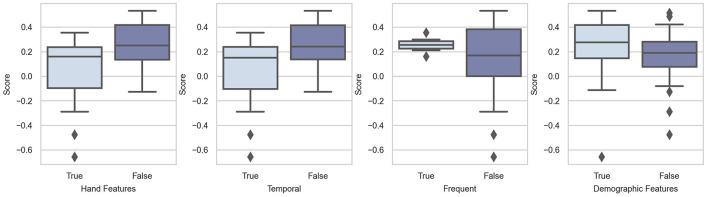
Box-plots of score distributions of feature engineering techniques.

We conducted the Mann-Whitney *U*-test to verify whether adding hand features is beneficial. However, the results were not satisfactory as per our expectations. On the contrary, the results showed that hand features have negative effects (*p* = 0.983). This may be because of the model architecture. Among the 15 teams that added hand features, 7 abandoned deep-learning methods and adopted traditional machine-learning methods, such as XGBoost (Wong et al., 2020). The model's inferiority may influence the results of the difference between adding and not adding hand features. We also conducted a hypothesis test on temporal features and frequent features, and the resulting *p*-values were 0.984 and 0.128, respectively. The results of the hypothesis test showed that temporal features are not helpful in improving the performance of the models. The addition of frequent features can yield better prediction results than that of temporal features. Figure 4 also supports our statistical results.

#### 3.3.2. Demographic Features

Demographic features, such as age and sex, have proven to be useful in ECG classification. Some cardiac diseases occur more frequently in specific patient subgroups. For example, ventricular fibrillation is predominantly observed in the aged (Iwami et al., 2003). Recent studies have shown that the difference between the chronological age and DNN-estimated age can be used as a predictor of mortality (Ladejobi et al., 2021; Lima et al., 2021). Although the features extracted from raw signals may include information related to these hand features, explicitly taking these hand features as input of models can help the model learn more knowledge than raw signals.

The statistical and graphic results proved our hypothesis that demographic features can help models make more accurate predictions. The *p*-value of the hypothesis test was 0.109. This means that adding demographic features is likely to be beneficial. As shown in Figure 4, the scores are relatively higher in the group with demographic features. In addition, we observe that the first and second teams and three other top-10 teams input demographic features to their models. We can, therefore, conclude that demographic features are helpful in the context of Challenge 2020.

### 3.4. Machine-Learning Models

In this section, we focus on the model architectures employed in Challenge 2020. We determined whether the teams used *basic machine-learning methods* or *DNNs*. We classified *DNNs* into three categories: *CNNs, recurrent neural networks (RNNs)/transformers*, and *attention mechanisms*.

#### 3.4.1. Basic Machine-Learning Methods

The basic machine-learning methods are all machine-learning techniques excluding DNNs, such as rule-based models and decision tree models. The most notable advantage of these models is that they are relatively easy to use compared with DNNs. Thus, these models can achieve good performance with less data, shorter training times, and lower computation resources. However, most of the time spent on traditional machine-learning methods is to extract features manually, requiring more intervention by specialists. In addition, the ECG data provided in Challenge 2020 were sufficient to support a more complex model (DNN architectures). Conventional methods may fit a large amount of data. In Challenge 2020, several teams adopted basic machine-learning methods, such as XGBoost (Uguz et al., 2020), random forest (Ignacio et al., 2020), and rule-based models (Smisek et al., 2020), whereas others combined these traditional methods with DNNs (Duan et al., 2020b; Zisou et al., 2020).

#### 3.4.2. DNNs

With the development of deep learning, we observe that most teams preferred to use DNN architectures. The prominent advantage of DNNs is that explicit feature extraction by human experts is not necessary, as features are automatically extracted by DNNs based on powerful learning ability and flexible design (Hong et al., 2020b). Related studies have shown that features extracted by DNNs are more informative (having higher importance scores than a random forest classifier) than hand features (Hong et al., 2017). The performance of deep-learning methods is also higher than that of traditional methods on many tasks, such as atrial fibrillation detection from single-lead ECG (Clifford et al., 2017) and sleep staging (Ghassemi et al., 2018). Therefore, the use of appropriate DNN architectures is of great significance.

**Figure 5:**
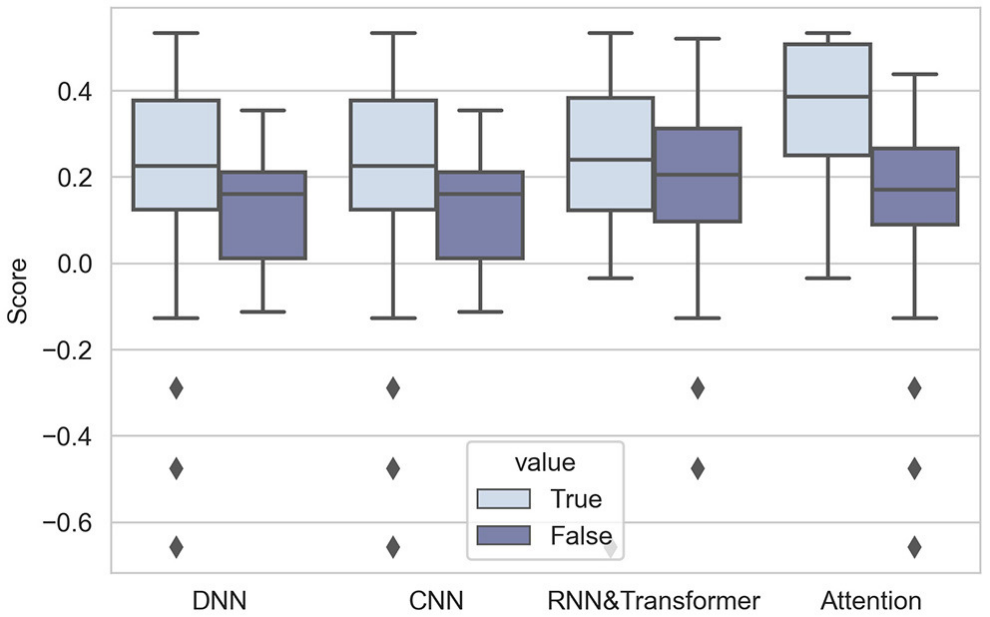
Box-plots of score distributions of machine-learning models.

As expected, the performance of DNNs was comparatively better in Challenge 2020. The 10 highest-ranking teams used DNNs, proving the popularity and effectiveness of DNNs. The first box figure in Figure 5 shows the performance of the DNN and non-DNN models, with the DNN models exhibiting higher scores.

The analysis of CNNs, RNNs or transformers, and attention mechanisms is presented as follows:

1. **CNNs**: CNN is one of the most popular DNN architecture that has been widely used in computer vision, signal processing, and natural language processing. The essential of the “convolutional” operation is local connectivity between two adjacent neural network layers, which makes it focus on the locality features while also reducing the model parameters (easier training). Such networks can automatically extract hierarchical representations relying on stacked trainable small convolutional filters (kernels). These filters can efficiently extract local representations and can reduce the complexity of models by sharing the same parameters in each layer. It is demonstrated that CNNs can capture more details in 12-lead ECG signals (Baloglu et al., 2019), so CNN is a proper choice as a feature extractor.
  It is notable that all DNNs used in Challenge 2020 include CNNs. Most of them employed a popular CNN architecture named ResNet (Residual Networks) (He et al., 2016a). The core component of ResNet is skip connections, which aims to solve the optimization degradation problem in the back-propagation process (as the network depth increases, accuracy gets saturated and degrades) (He et al., 2016b). In Challenge 2020, the results are in accord with the general point of view–using CNNs can significantly improve the performance, as shown in the second group of box-plots of Figure 5.
2. **RNNs/transformers**: In addition to CNN, RNNs and Transformer (Vaswani et al., 2017) are also widely used DNNs, especially for sequential data, such as time series, event sequences and natural language (Hong et al., 2020b). RNNs take the output from the previous step as input and iteratively update hidden states and memory. Transformer adds attention to sequential modeling and allows sequences to be parallel processed. Different from CNNs, RNNs and transformers mainly focus on temporal dependency rather than local representation. Another advantage is that RNNs and Transformer can handle inputs of various lengths, which is also sometimes necessary for time series data.
  Some teams combine two kinds of architectures in Challenge 2020 (Fayyazifar et al., 2020; Hasani et al., 2020; Natarajan et al., 2020; Oppelt et al., 2020) by applying an RNN or Transformer on representations obtained by CNNs. This is commonly preferred for long ECG signals, because combining two kinds of DNNs can both extract local features and summarize features along the time dimension to obtain global representations. From the third box figure in Figure 5, we can see that RNNs and Transformers can help improve the performance of models.
3. **Attention mechanism**: Because of the emergence of the Transformer, attention becomes a widely used mechanism in DNN architectures. The attention mechanism is essentially a kind of weighted sum, and we categorize it into two classes: position-wise attention and channel-wise attention. In detail, we see Transformer as position-wise attention, because Transformer assigns different weights for features extracted from different time points. In addition, we see squeeze-and-excitation block (Hu et al., 2018) as channel-wise attention, because SE block produces weights for each channel of input features. These two kinds of attention mechanisms both have characteristics of plug and play, which means they can easily be combined with DNN models. By applying attention, models can focus on key time steps of long time series (position-wise), or more informative channels (channel-wise attention).
  The results are notable: 4 highest-ranking teams all add the attention mechanism to their models, showing the prevalence of attention. The result of the Mann-Whitney *U*-test also proves that attention can improve the performance of models (*p*-value is 0.0059, less than 0.01). The effect of using attention is intuitively shown in the fourth box figure in Figure 5.

### 3.5. Training Strategy

In this section, we analyze three aspects of the model: *model ensemble, end-to-end*, and *multi-binary classification*.

#### 3.5.1. End-to-End

The end-to-end model takes raw data as input and outputs the target directly, without considering how the features are generated or what they represent. During the process of training end-to-end models, less supervision is required, making it more applicable in the real world. Non-end-to-end models divide the whole task into several sub-tasks, indicating that different sub-tasks may not be consistent and the gap between them may result in non-optimal performance. In addition, when the model is divided into multiple parts, the errors of each part may accumulate and propagate into the next stage. In contrast, end-to-end training can provide more space for models to adjust themselves depending on the input data, making models fit the data better. However, the interpretability of the end-to-end model is always a critical question, especially for medical purposes. Without knowing how the model makes decisions, the results may be unreasonable to be accepted by clinicians and difficult to verify.

**Figure 6:**
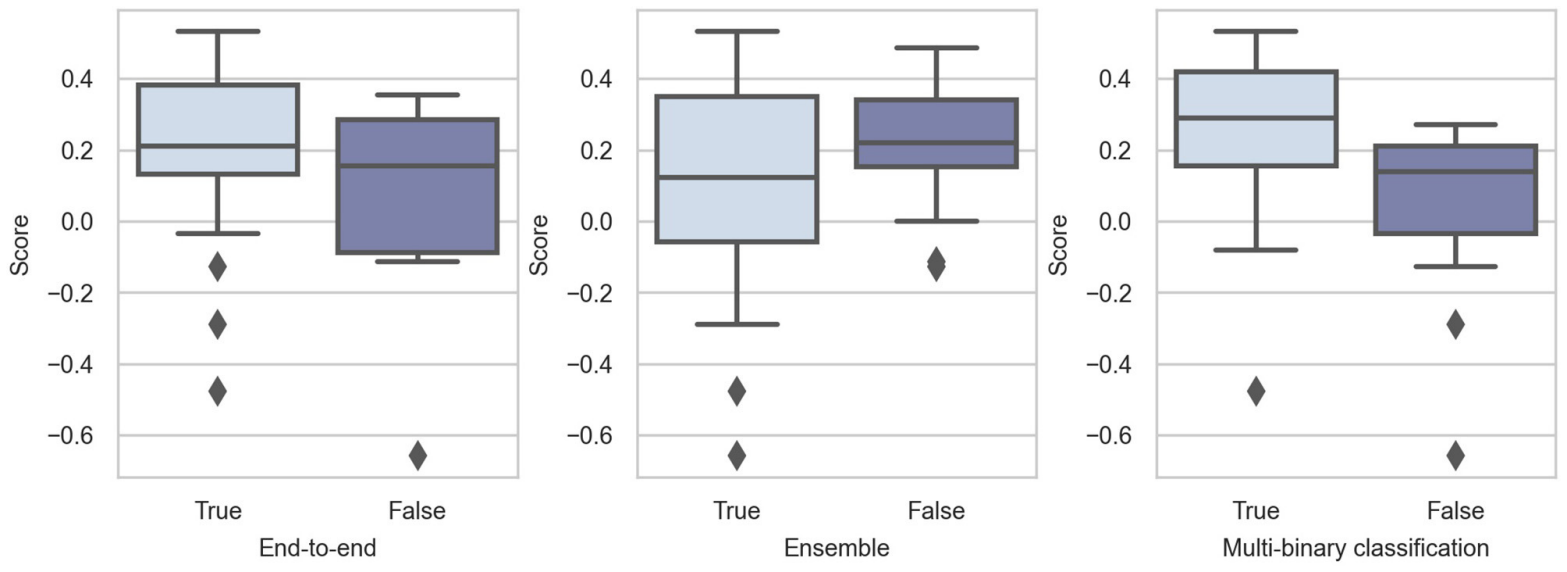
Box-plots of score distributions of training strategy techniques.

In Challenge 2020, the top-10 teams adopted end-to-end models, showing the popularity of such models. As shown in Figure 6, these models perform considerably better than non-end-to-end models. The effect and popularity of end-to-end models in Challenge 2020 were related to DNNs, because most DNNs are structured in this manner.

#### 3.5.2. Multi-Binary Classification

A multi-label classification problem can be solved as a multi-class problem directly or a combination of multiple binary classification problems. In detail, multi-binary classification means training a binary classifier for each class to decide whether the sample belongs to this class. This means that the predictive possibility of each class is independent. This is advantageous for training because the multi-class task can be divided into several simple binary classification tasks. However, this neglects the relationship between different diseases, which may have negative effects. In contrast, the output of the multi-class problem is only a vector representing the predictive possibilities, and the sum of these is 1. This is a relatively difficult task compared with the multi-binary classification.

Whether using multi-binary classification significantly influences the performance. The *p*-value of the Mann-Whitney *U*-test is 0.0018, indicating that using multi-binary classification can significantly improve the performance of the classifiers. Consequently, we believe that the relationship between diseases is not very important. For such a difficult multi-class task, multi-binary classification can reduce the difficulty of training and help achieve better performance.

#### 3.5.3. Model Ensemble

A model ensemble is a learning paradigm that combines multiple learners to improve the overall performance. The commonly used ensemble methods include *bagging* (average predictions or votes for one prediction) and *boosting* (weighted bagging). The core concept of bagging is to average the predictions of several models or make predictions according to the majority vote. Boosting can be regarded as a type of weighted bagging because the classifiers are assigned different weights. Bagging and boosting can improve the performance of the ensemble model by reducing the error caused by the variance and bias, respectively. The motivations behind the ensemble used in Challenge 2020 were mainly to combine models designed for different features or to enhance the ensemble model by combining several models. In addition, bagging was the most commonly used ensemble method in Challenge 2020.

We notice that the three highest-ranking teams used the model ensemble (Natarajan et al., 2020; Zhao et al., 2020; Zhu et al., 2020), but only 14 out of 41 teams employed this strategy. The results are not expected, and we believe that this is because the model ensemble can help improve the single model, whereas it is less meaningful to compare among different teams.

### 3.6. Applications to the Real World

In this section, we consider some techniques that are necessary in real-world scenario but rare in Challenge 2020, which are *post-processing, interpretability, unknown classes* and *unseen patients*.

#### 3.6.1. Post-processing

We exclude ensemble methods and threshold optimization in this section because they are mentioned in the previous sections. Except for these two types of post-processing techniques, we find that only one team performed hard sample mining (Chen et al., 2020) as a post-processing technique. Based on the same idea as hard sample mining, some techniques (Orphanidou et al., 2014) can be used to detect and remove low-quality ECG segments (hard samples). The summarized results for high-quality ECG segments are more believable. Finally, the interactions between labels can be considered to post-process the predictions. For example, the reward matrix in Challenge 2020 (see Figure 2 in Alday et al., 2020) indicates that class labels are correlated with each other. In this situation, predicting one label might help predict another correlated label.

#### 3.6.2. Interpretability

The lack of model interpretability is a critical problem for machine-learning models, especially for deep-learning-based models. In Challenge 2020, only two teams (Raipal et al., 2020; Żyliński and Cybulski, 2020) showed feature importance in interpreting the model. The factors that lead to model predictions are unknown for clinicians. Here, we discuss two potential directions for improving the interpretability of the models.

Uncertainty represents how “certain” a model is of each prediction it generates. Although it is difficult to obtain statistical guarantees on performance (which requires true data distribution), estimating the level of uncertainty of predictions is more important than improving accuracy for clinicians (Tonekaboni et al., 2019). A common method for estimating the uncertainty of DNNs with dropout is Monte Carlo dropout (Gal and Ghahramani, 2016). This technique uses dropout during inference and applies the model on the same input multiple times to sample many outputs. In the real world, uncertainty can help clinicians to determine the degree of model reliability, as high uncertainty in ECG classification strongly corresponds with a low diagnostic agreement with the interpretation of the cardiologist (Vranken et al., 2021).

The relative importance is visualized, as clinicians view ECG signals as figures rather than numbers, unlike what deep-learning engineers do. Consequently, highlighting the important component of an ECG segment is vital for the interpretability of the models. The relative importance of different components obtained by models should be evaluated to examine the evidence of the results in a way that cardiologists can understand. Thus, the models can “explain” their predictions, while identifying more details that may be neglected by humans (Elul et al., 2021). The methods for achieving this goal include spectro-temporal attention (Elul et al., 2021) and layer-wise relevance propagation (Binder et al., 2016). These methods emphasize the more important part of the ECG signal on figures to help humans understand what the models care about the most.

In summary, interpretability is necessary for ECG signals in real-world scenarios, and it requires more attention. Researchers can attempt to explain why the models produce their predictions, and this can prompt the real-world application of automated ECG interpretation.

#### 3.6.3. Unknown Classes and Unseen Patients

The classification model is trained on a limited range of datasets, but it is used on an unlimited range of data in the real world. A team in Challenge 2020 considered the differences among databases and employed domain adversarial training (Hasani et al., 2020). However, this team neglected individual-level differences. In a real-world scenario, there are numerous unknown classes and unseen patients. To solve this problem, the models for automated ECG interpretation should have the ability to quickly adapt to unknown classes and unseen patients.

For unknown classes, the model needs to 1) automatically detect whether there is an unknown class and 2) rapidly adapt to the unknown class. To achieve the first goal, we can decouple the multi-class classification task into multiple binary classification tasks and add new classification heads to meet the new rhythm types. If all the existing prediction heads output “False,” it indicates that we have met an unknown rhythm type. Other techniques related to the open world (Bendale and Boult, 2015) are also useful. To achieve the second goal, an effective method is to build a separate task-specific simple machine-learning model on top of existing engineered features, such re-training only the final fully connected layer in DNNs, while maintaining the other weights.

A more difficult problem is the gap in the data distributions among different patients. This gap is caused by not only the physiological differences but also other factors such as medical devices and data storage formats (Elul et al., 2021). In this situation, the model trained on existing data might not work equally well on unseen patients. There are many noteworthy attempts to overcome this problem, such as meta-learning to find a set of easily generalized initial parameters (Banluesombatkul et al., 2020) and employing regularization on the loss function (Elul et al., 2021). These methods can benefit the performance on unseen patients and may be helpful on existing patients.

Thus, a good model is not the best on the training set, but the best on the unseen dataset. Thus, how to tackle the “unseen” problems is the key for machine-learning models.

## 4. Discussion

In this section, we summarize and discuss the five most influential and interesting practical points based on previous results.

### 4.1. Data Augmentation Should Be Employed and Adapted to Specific Scenarios

It is universally accepted that increasing the amount of training data contributes to the improvement of deep-learning-based models. However, high-quality labeled data are limited in 12-lead ECG classification tasks. Data augmentation by generating synthetic patterns is a model-agnostic solution to this problem.

In addition to cropping, introducing external data, and adding noise, methods based on random transformations, such as flipping, window warping, and masking, are commonly used for the augmentation of time-series data. However, we notice that no method based on the time-frequency domain or frequency domain alone was used for Challenge 2020. In recent years, data augmentation from these two perspectives has drawn considerable attention in many fields (Lee et al., 2019; Park et al., 2019; Gao et al., 2020), including ECG classification tasks. Moreover, handling the severe class imbalance problem in ECG through data augmentation can be a future research direction.

Furthermore, choosing the most appropriate augmentation method remains a challenge. Although the authors in Iwana and Uchida (2021) discussed the advantages and disadvantages of various methods and offered suggestions for using different time-series data types, the effectiveness of various augmentation methods is still based on empirical experiences and experiments.

### 4.2. Combining Different Features Can Improve Performance

To fully utilize expert knowledge and metadata beyond raw signals, traditional features (not from deep-learning models) are commonly applied for tasks in the medical field (Supratak et al., 2016; Hong et al., 2017, 2019b). In Challenge 2020, teams not only used demographic features such as sex and age but also extracted signal-specific features using traditional methods. In terms of integration, most teams combined traditional-method-based features and deep-learning-based features using simple concatenation. In this way, models can learn extra information from traditional features and retain the generalization ability of deep features (Cheng et al., 2016; Natarajan et al., 2020).

However, most teams neglected a simple technique: hand feature interaction. DeepFM (Guo et al., 2017) provides an accessible solution to this problem, by adding an interaction technique to wide and deep architectures (Cheng et al., 2016). In addition, how to achieve “feature fusion” is a potential direction for better combining the two types of features. The outer product is another universally employed method (Gao et al., 2016; Yu et al., 2017).

### 4.3. A Hybrid Design of Different Types of DNNs Is Better Than Using a Single Type

First, deep-learning models prevailed in Challenge 2020. As shown in Table 2, 82.93% of the teams select DNNs as their models or part of their models. Some teams that use relatively simple models, such as rule-based models (Smisek et al., 2020), achieve good scores. We believe that DNNs exhibit a higher performance only when the model is suitable and the data are well-preprocessed. In addition, combining raw data and domain knowledge is a critical problem in deep-learning-based methods.

Second, the choice of the DNN type is essential. In Challenge 2020, CNN-based models were dominant: all DNNs were CNNs or extracted features from CNNs, indicating that CNNs may be a better choice when latent representations are extracted from raw ECG signals. However, RNNs or transformers can also be applied to discover the temporal dependency of the representations obtained by CNNs. This was a common way to combine CNNs and RNNs in Challenge 2020 (Hasani et al., 2020; Natarajan et al., 2020; Oppelt et al., 2020) and was adopted by three of the top-5 teams.

Finally, we want to emphasize the attention mechanism because of its significant performance improvement. The top-3 teams in Challenge 2020 used the attention mechanism, which can be classified as channel-wise attention (squeeze-and-excitation) and location-wise attention (transformers). The former assigns different weights to each channel (channels can be implicit in each layer of DNNs), and the latter assigns different weights to the representation vectors in each time step. Overall, both types of attention make models learn to recognize more useful information. We can easily add the attention mechanism to the designed models as a plugin block, and this has proven to be beneficial, as described in Section 3.4.2.

When an appropriate model for ECG tasks is constructed, the advantages of different base learners should be combined to design the most powerful model.

### 4.4. The Use of End-to-End Architectures Should Depend on the Task Being Solved

End-to-end models are becoming increasingly popular owing to the development of DNNs because they do not require significant manual interference, reducing the cost and time consumed in automated ECG interpretation.

However, although end-to-end models are attractive, some limitations exist. First, the performance of each part of the entire model cannot be quantified. Thus, each component of the whole model is designed empirically without any separable and measurable feedback, thereby causing difficulty in the modification of the model. Second, end-to-end models are much slower to be trained compared with decomposition methods because the gradients are noisier and less informative, as demonstrated theoretically and empirically (Shalev-Shwartz et al., 2017). Third, end-to-end models are less flexible because we cannot process the features generated in the middle layers.

Overall, it remains unclear whether to use end-to-end models, depending on the scenario and domain knowledge.

### 4.5. Multiple Models Are Better Than One

The model ensemble is a model-agnostic and efficient paradigm for improving the performance of a single model. A method from Challenge 2017 showed that the ensemble classifier outperformed single models (Hong et al., 2017). The most common ensemble methods included bagging (bootstrap aggregation), boosting, and stacking.

- Bagging is an ensemble method that trains base learners from different bootstrap samples (subsampling with replacement for the training data). Bagging is more efficient because the base models can be trained in parallel. We regard most ensemble models in Challenge 2020 as being obtained by bagging because they are trained in parallel. However, strictly speaking, they are different from bagging because their sampling methods include not only bootstrap but also other methods.
- Boosting is a family of methods that train models in order, with each base learner relying on the last one. For example, the incorrectly classified samples from the last base learner may be assigned a higher weight in the current training process to emphasize its importance. This is generally employed in decision tree models, such as AdaBoost (Freund and Schapire, 1997) and XGBoost (Chen and Guestrin, 2016).
- Stacking trains first-level learners by using training data and then takes the output from the first-level learners together with the training labels to train a second-level learner. In this way, all first-level learners are combined, and the second-level learner produces the prediction.

There is no conclusion about which ensemble method has the best performance among the three most common ensemble models. However, it is incorrect that more base learners lead to a better performance (Zhou et al., 2002). In other words, composing an ensemble with a part of the base learners instead of the whole set is more appropriate.

## 5. Conclusion

In this study, we collected 41 methods used in Challenge 2020 and conducted a meta-analysis on them, focusing on the aspects of data preprocessing, feature engineering, machine-learning models, training strategy, and applications to the real world. We statistically analyzed and visualized the effectiveness of each technique. We then discussed the advantages and disadvantages of the techniques in terms of the aforementioned aspects. Finally, we summarized five practical lessons based on the analysis, providing practical and instructive experiences in cardiac disease classification tasks based on ECG.

## Data Availability Statement

The original contributions presented in the study are included in the article/Supplementary Material, further inquiries can be directed to the corresponding authors.

## Author Contributions

SH: conceptualization. SH and WZ: methodology and writing-original draft. WZ: visualization. All authors contributed to data extraction, data interpretation, reviewing, editing, and approval of the final version.

## Funding

This work was supported by the National Natural Science Foundation of China (nos. 62102008 and 62172018) and the National Key Research and Development Program of China under grant no. 2021YFE0205300.

## Conflict of Interest

The authors declare that the research was conducted in the absence of any commercial or financial relationships that could be construed as a potential conflict of interest.

## Publisher's Note

All claims expressed in this article are solely those of the authors and do not necessarily represent those of their affiliated organizations, or those of the publisher, the editors and the reviewers. Any product that may be evaluated in this article, or claim that may be made by its manufacturer, is not guaranteed or endorsed by the publisher.

## Footnotes

^1^ https://www.cinc.org/archives/2020/

^2^ https://www.scipy.org

## Abbreviations

Challenge 2020: PhysioNet/Computing in Cardiology Challenge 2020
CinC: Computing in Cardiology
CNN: Convolutional Neural Network
DNN: Deep Neural Network
ECG: Electrocardiogram
RNN: Recurrent Neural Network.
